# Barriers beyond behavior: structural determinants of behavioral health treatment disparities among Hispanics in the U.S.

**DOI:** 10.3389/fpubh.2026.1733641

**Published:** 2026-04-24

**Authors:** Marissa Gonzalez, Jose Eduardo Cabrero Castro, Emre Umucu, Alfonso Rojas-Alvarez

**Affiliations:** Department of Public Health Sciences, The University of Texas at El Paso, El Paso, TX, United States

**Keywords:** health policy, Hispanic health, structural barriers, substance use disorders, treatment disparities

## Abstract

**Background:**

Persistent disparities in substance use treatment access undermine efforts to achieve equitable public health outcomes in the United States. Hispanic populations, who report lower prevalence of substance use, nevertheless face systemic barriers to care. This study examines how socioeconomic status, insurance coverage, and ethnicity influence treatment utilization and perceived barriers, with attention to structural and sociocultural factors shaping disparities.

**Methods:**

This cross-sectional study analyzed data from the 2023 National Survey on Drug Use and Health (*N* = 56,705). Logistic regression assessed associations between ethnicity, insurance coverage, substance use, and treatment utilization, adjusting for demographic and socioeconomic covariates.

**Results:**

Hispanic respondents had significantly lower odds of reporting SUD (OR = 0.68), illicit drug use (OR = 0.68), and alcohol use disorder (OR = 0.78), and were less likely to receive behavioral health treatment (OR = 0.67) or medication-assisted treatment (OR = 0.59). Structural barriers, notably lack of insurance (47%) and high costs (40%), surpassed stigma-related barriers among Hispanics. While Medicaid (OR = 1.14) is positively associated with treatment access compared to being uninsured, substantial gaps persisted relative to private insurance.

**Conclusion:**

Structural policy reforms emphasizing expanded insurance access, affordable treatments, and culturally tailored harm reduction strategies are critical to addressing disparities. Health education and targeted community interventions may be able to effectively overcome systemic barriers to behavioral health care for Hispanic communities.

## Introduction

Substance Use Disorders (SUDs) remain a persistent and devastating public health crisis in the United States, with profound implications for morbidity, mortality, and healthcare costs. In 2023, over 48.5 million Americans met the criteria for SUD, yet only 15% received treatment, reflecting a stark care gap (1). Since 2000, drug overdoses have claimed approximately one million lives, with 107,941 deaths in 2022 alone (2). Drug overdose now ranks as a leading cause of death, driven largely by synthetic opioids such as fentanyl (3). Previous studies have found the opioid epidemic alone accounted for 3.1 million years of life lost in 2022 (4) and a cumulative economic cost of $1.5 trillion (5).

While these statistics underscore a national crisis, the burden is not shared equally. A critical paradox defines the Hispanic experience in behavioral health: while Hispanic populations frequently demonstrate lower overall prevalence of SUD compared to non-Hispanic Whites, they face a disproportionately wider treatment gaps and significantly poorer outcomes when care is delayed (6). This disparity suggests that lower utilization rates among Hispanics are not merely a function of lower need, but the result of systemic exclusion. While national policies have aimed to expand coverage and enhanced parity in mental health services, persistent disparities, rooted in structural barriers like limited insurance, stigma, geographic inaccessibility, and punitive policies, undermine equitable treatment access and outcomes (7–9).

Amid this crisis, health insurance plays a critical role in behavioral health care access by enabling preventive services and harm reduction strategies, both of which are essential for reducing overdose risk and supporting sustained recovery. Historically, uninsurance rates are disproportionately higher among people who use drugs, which impacts treatment seeking behaviors (10). Individuals with comprehensive coverage are significantly more likely to initiate and remain engaged in treatment, while the uninsured or underinsured face persistent barriers, including prohibitive costs, limited provider networks, and inconsistent care (11, 12).

To address these gaps, federal reforms such as the Mental Health Parity and Addiction Equity Act (70), the Affordable Care Act (69), and state-level Medicaid expansions have sought to improve access to behavioral health care, particularly for low-income individuals. These policies have helped reduce the uninsured rate, increase utilization of evidence-based treatments such as medication-assisted treatment (MAT), and extend coverage, particularly through Medicaid, to millions of previously underserved individuals (13, 14). However, disparities remain between insurance types. Private insurers generally offer broader provider networks, higher reimbursement rates, and more consistent access to specialized care and MAT compared to public plans (15, 16).

Facility participation rates, defined as the percentage of treatment facilities that accept a given insurance type, vary considerably by insurance type, with acceptance rates highest for private insurance (approximately 66%−76%) and Medicaid (55%−75%), while notably lower for Medicare (34%−47%), Tricare (41%), and the Indian Health Service (12%) (17). Even where coverage exists, practical barriers such as long wait times, limited MAT availability, and insufficient provider capacity impede access (15). Publicly insured patients also report higher levels of perceived provider discrimination, reduced care quality, and longer wait times compared to their privately insured counterparts (18, 19). This is especially true in states that did not expand Medicaid coverage under the Affordable Care Act, where emergency departments often serve as default treatment sites (13).

### Racial and ethnic disparities

#### Differences across demographics

Socioeconomic status is a critical determinant of SUD risk and access to behavioral health care. Medicaid enrollment, often a proxy for low income, correlates with higher overdose mortality and forgone treatment needs (20). Structural drivers like poverty, unemployment, housing insecurity, and limited education hinder prevention and recovery (21–23), while income inequality and health illiteracy further delay problem recognition and treatment initiation (24, 25).

Racial and ethnic disparities compound these inequities. Since 2015, overdose mortality has risen sharply among Black, Indigenous, and Latino communities, surpassing rates among White individuals for the first time in 2020 (26). Between 2015 and 2019, opioid overdose deaths rose by 433% for Latino youth and 361% for Black youth (27). These outcomes are exacerbated by lower treatment utilization and completion rates (28, 29) along with persistent disparities in access to medication-assisted treatment among Black, Indigenous and Latino populations. Even after an overdose, Black and Hispanic patients are less likely to receive MAT than their White counterparts (30, 31). Structural barriers such as residential segregation and the uneven geographic distribution of MAT facilities, entrench these inequities, leaving communities of color with limited access to life-saving treatment options (32).

Disparities are further stratified by gender. Black and Hispanic women are significantly less likely to engage in care; studies estimate that 87% of Black women and 81% of Hispanic women forego necessary treatment, compared to 68% of White women (33). Contributing factors include economic hardship, limited childcare options, and lack of culturally competent providers (9, 34). Addressing these inequities requires systemic efforts to confront the structural roots of unequal care access and outcomes.

Hispanic populations, who generally have lower SUD prevalence, experience significant variations in treatment access and outcomes. Nearly 91% of Hispanics experiencing a SUD did not receive the necessary treatment at a specialty facility (35). When care is sought, Hispanics face more delays in accessing treatment and demonstrate poorer outcomes compared to non-Hispanics (6, 29, 36). Poverty, underinsurance, limited healthcare access, low educational attainment, language barriers, and uncertain legal status produce a complex ecosystem of treatment barriers that undermine prevention and recovery efforts in Hispanic communities (8, 9, 37, 38).

#### Systemic and structural barriers

Understanding behavioral health inequities among Hispanic populations requires analyzing structural determinants as the drivers of social determinants of health. While social determinants encompass the conditions of daily life, they are fundamentally shaped by structural determinants, which represent the upstream macroeconomic and political mechanisms, such as governance and social class, that generate stratification and dictate the unequal distribution of resources (39). In the context of Hispanic health, these disparities are compounded by broader sociopolitical forces, including racialized drug narratives, restrictive immigration enforcement, and underinvestment in health services among Hispanic communities (40). Although Hispanic individuals constitute only 19.5% of the U.S. population (41), they are disproportionately represented in federal prosecutions, comprising 49.8% of all cases in 2024, including 45% of drug trafficking and 94% of immigration-related offenses. This disproportionate criminalization illustrates how racialized drug narratives and punitive policy environments shape risk exposure and reinforce structural inequities. Hispanic defendants also face harsher and longer sentencing compared to White defendants (42), further entrenching cycles of criminalization and marginalization.

Immigration status functions not merely as a demographic variable but as a critical determinant that legally dictates eligibility for the social safety net (43). Restrictive immigration enforcement leads to exclusionary policies, specifically those barring undocumented immigrants and many lawful permanent residents from federally funded programs like Medicaid and the Affordable Care Act, serve as institutional barriers to care (68). This statutory exclusion creates a “chilling effect,” where fears of deportation or “public charge” determinations lead individuals to forego necessary behavioral health treatment, even when eligible (44). Furthermore, these policies produce “spillover effects” that increase psychological distress and lower health system engagement even among US-born Latinos in mixed-status families (45, 71). Consequently, immigration policy restricts access to healthcare through two simultaneous pathways: limiting the supply of care via insurance exclusion and suppressing demand through criminalization and stigma (46).

#### Culture and heterogeneity

Cultural and attitudinal stigma including fears of shame, familial rejection, or reputational harm, further suppresses treatment-seeking behaviors among Hispanic communities (7, 8, 47). Distrust in healthcare, skepticism toward treatment efficacy, and opposition to abstinence-only models add to the reluctance (7, 8). Still, protective cultural factors exist. Research suggests that *familismo*, religious involvement, and conservative attitudes have been linked to lower substance use, particularly among first-generation immigrants (47–49).

Finally, it is critical to acknowledge that the Hispanic population is not a monolith; rather, it is composed of diverse subgroups with distinct migration histories and sociocultural contexts. The literature reveals substantial heterogeneity in outcomes driven by these specific histories. For instance, Puerto Ricans exhibit a significantly higher prevalence of mental health and substance use disorders compared to other Hispanics (1). This subgroup also demonstrates a reversal of the “immigrant paradox,” wherein island-born individuals face significantly higher overdose mortality than those born in the U.S. (50). For other subgroups, acculturation factors like being U.S.-born, English-proficient, or third-generation are associated with increased rates of overall psychiatric disorders and substance use disorders, while foreign-born status often confers a protective effect (51). Treatment-seeking behaviors are differentially stratified by both ethnicity and legal determinants; while Puerto Ricans generally have higher access to medical sectors due to citizenship status, prohibitive barriers related to legal status severely inhibit treatment access for other subgroups (29).

### Theoretical framework and hypotheses

**Figure 1:**
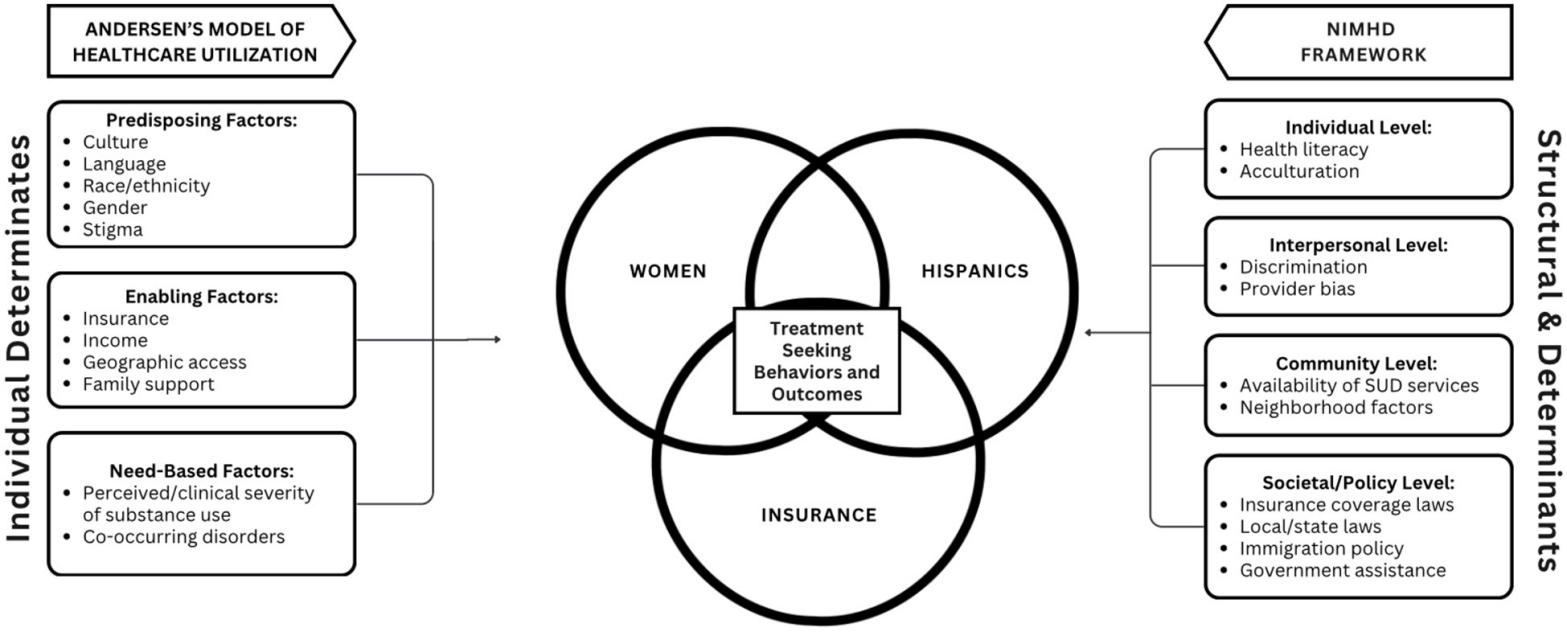
Analytical framework on the intersection of gender, ethnicity, and insurance. This figure illustrates how this study integrates the Andersen Behavioral Model and the NIMHD Research Framework. Andersen's model emphasizes predisposing characteristics, enabling resources, and need, which guide treatment use. The NIMHD framework incorporates five domains, biological, behavioral, physical/built environment, sociocultural environment, and health care system, across four levels of influence (individual, interpersonal, community, societal. The study primarily operationalizes individual-level sociocultural factors (for example, ethnicity, stigma-related perceptions) and health-care system factors (for example, insurance, cost, access) consistent with both frameworks. Sex/gender is treated as a predisposing characteristic at the individual level.

This study draws on two complementary perspectives: the Andersen Behavioral Model of Health Services Use and the NIMHD Minority Health and Health Disparities Research Framework (Figure 1). Andersen's model explains treatment utilization as a function of predisposing factors (such as age, sex, ethnicity, education), enabling resources (such as insurance coverage, income, and other material resources), and clinical need. The NIMHD framework situates these determinants within five domains (biological, behavioral, physical/built environment, sociocultural environment, and the health care system) and across four levels of influence (individual, interpersonal, community, and societal), providing a multilevel lens for understanding health disparities.

In this study, the two models align in useful ways. Predisposing characteristics correspond to sociocultural factors at the individual level; enabling resources reflect access points within the health care system; and need reflects behavioral/biological indicators of clinical status. These frameworks guided our analytic choices: treatment models were conditioned on need, enabling resources were included to assess structural access constraints, and perceived barriers were examined as expressions of sociocultural and system-level influences.

To clarify the conceptual-to-empirical linkage, the core constructs are represented as follows:

- Predisposing factors: Hispanic identification, age, sex, education.
- Enabling resources: insurance type (uninsured, private, Medicaid/Medicare, military), income, government assistance, and metro/non-metro residence.
- Need: AMI/SUD criteria status.
- Use/outcomes: receipt of AMI/SUD treatment, MAT use, and substance use behaviors.
- Perceived barriers: cost, lack of coverage, logistical challenges, stigma, and treatment-related beliefs.

### Hypotheses

Guided by these frameworks, we test the following hypotheses:

*H1*. Hispanic adults will have lower odds of meeting criteria for AMI/SUD, alcohol use disorder, and past-year illicit drug use than non-Hispanic adults.
*H2*. Uninsured adults will have lower odds of receiving past-year AMI/SUD treatment than insured adults.
*H3*. Hispanic adults will have lower odds of receiving past-year AMI/SUD treatment, both among those meeting AMI/SUD criteria and those not meeting criteria.
*H4*. Among adults with unmet or forgone care, Hispanic adults will be more likely to report structural or economic barriers (such as cost or coverage) than attitudinal barriers.
*H5*. Public insurance types will differ from private insurance in their association with treatment utilization and reported structural barriers.

## Materials and methods

### Data source

For this study, we use the National Survey on Drug Use and Health (NSDUH) collected by the Substance Abuse and Mental Health Services Administration (67). This dataset, collected annually, provides nationally representative data on the use of tobacco, alcohol, drugs, substance use disorders, mental health issues, and the receipt of services for these conditions among the population aged 12 and older in the United States. For the 2023 cross-section (*n* = 56,705), a multistage area probability sampling strategy was followed, using in-person and web-based questionnaires to collect data. We analyzed treatment utilization across the full civilian, non-institutionalized U.S. population rather than restricting the sample to individuals with AMI or SUD to ensure nationally representative estimates and capture barriers among those with perceived needs who may not meet diagnostic thresholds. All NSDUH measures are based on self-report, which may introduce recall and social desirability bias, especially for sensitive topics such as substance use and treatment attitudes. This approach aligns with prior NSDUH-based studies examining access and utilization patterns in the general population (52, 53).

### Dependent variables

We organize the analysis using three groups of dependent variables. The first group includes binary indicators capturing whether respondents reported experiencing AMI or SUD in the past year, and whether they received treatment for either condition in the past year, which operationalizes the concept of treatment utilization. The AMI/SUD variable is a composite indicator identifying adult respondents (aged 18 or older) who met *DSM-5* criteria for AMI or SUD within the past year. The treatment variable is a recoded binary indicator reflecting whether respondents received either substance use treatment or mental health treatment. The second group concerns binary measures of health behaviors such as self-reported alcohol use disorder, medication-assisted treatment (MAT) utilization, and illicit drug use in the last year.

The final set of variables captures reasons for forgoing care for AMI or SUD, grouped into two domains: (1) structural or socioeconomic barriers, and (2) attitudinal or stigma-related barriers. Structural barriers included lack of insurance, insufficient coverage, high costs, logistical issues (e.g., transportation, childcare, or appointment times), and having sought but not received treatment. Attitudinal or stigma-related barriers included “*concerns family, friends or religious group wouldn't like it*,” “*they didn't think it'd work*,” and “*worries what people would think*.”

### Independent variables

Our key independent variable is ethnicity, specifically whether the respondent identifies as Hispanic. Ethnicity was conceptualized as a self-reported social construct capturing cultural heritage rather than a biological category. We utilized the NSDUH's binary indicator, which classifies respondents as Hispanic if they self-identified as being of “Hispanic, Latino, or Spanish origin or descent” regardless of race. While this aggregation limits the ability to detect subgroup-specific variations (e.g., between Mexican and Puerto Rican populations), it was necessary to maintain statistical power. Hispanic identity is the exposure of interest for disparity analyses. Age and sex were treated as pre-exposure demographic standardizers. Enabling-resource variables, insurance type, educational attainment, income, employment, government assistance, and metro/non-metro residence, were conceptualized as potential mediators of the association between Hispanic identity and behavioral health outcomes, consistent with Andersen's “enabling resources” domain. In models where insurance was the focal exposure (enabling resource), Hispanic identity was included as a covariate to account for confounding of insurance–outcome associations.

### Analytical approach

To account for the complex, multi-stage sampling design of the NSDUH, all statistical analyses were conducted using the “*svy”* command suite in Stata. Before analysis, the data were structured using the “*svyset”* command to incorporate the person-level analysis weights (WTSY23), stratification variables (VESTR), and primary sampling units or clusters (VEREP) provided in the 2023 Public Use File. This procedure utilizes Taylor series linearization for variance estimation, ensuring that all reported standard errors and *p-values* correctly reflect the clustering and stratification of the survey design. Weighted analyses were used for all descriptive characteristics and multivariable models to produce accurate population-level estimates. All weighting for our analysis was done following the NSDUH Public Use File Data User's Guide (54, 55).

**Figure 2:**
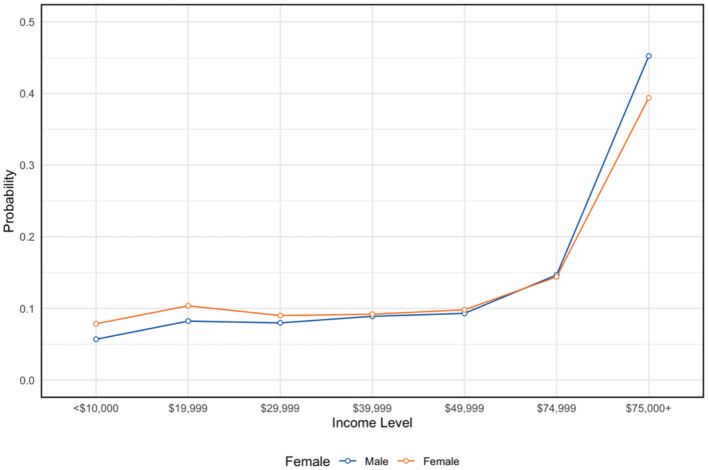
Income disparities by gender. NSDUH sample, 2023 (*n* = 56,705). Y-axis represents the percent distribution of each group.

**Figure 3:**
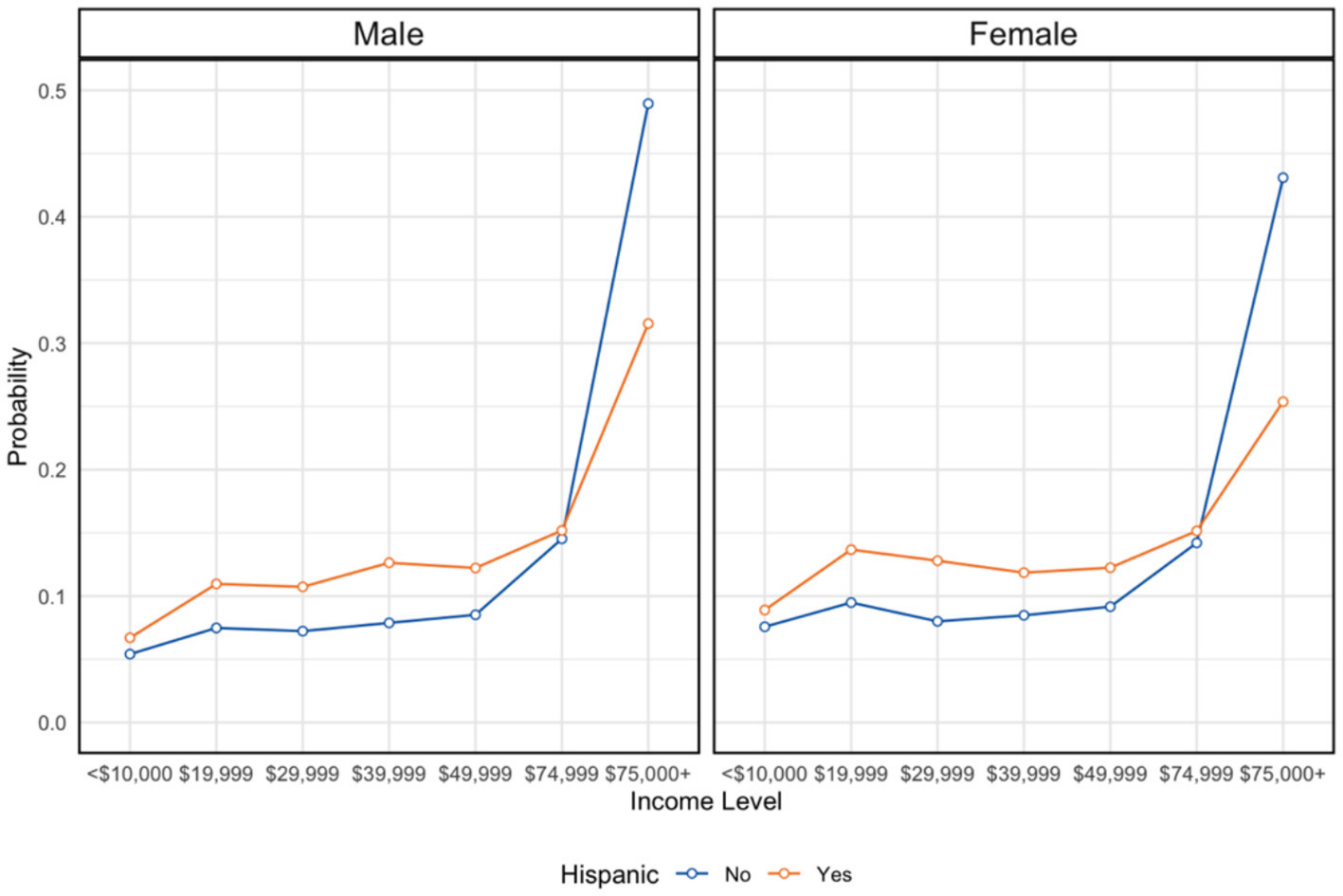
Income and gender disparities by Hispanic identification. NSDUH sample, 2023 (*n* = 56,705). Y-axis represents the percent distribution of each group.

First, we first generated survey-weighted descriptive characteristics for the full analytic sample (*N* = 56,705), overall and stratified by Hispanic identification (Table 1). For categorical variables, we report weighted percentages and standard errors. We also include unweighted sample sizes for reference. Group differences by ethnicity were evaluated using design-adjusted tests appropriate for complex samples (χ^2^ / bivariate regression estimates implemented by Stata's svy commands). For each characteristic, corresponding *p-values* in Table 1 reflect these survey-adjusted comparisons using a bivariate regression estimation, as the preferred method in Stata for weighted comparisons. We then estimated a series of survey-weighted logistic regression models to examine associations of Hispanic ethnicity and insurance coverage with behavioral health outcomes and perceived barriers (Tables 2–5). For all models, coefficients are presented as odds ratios (ORs) with design-adjusted standard errors (SEs). Each model included Hispanic identification as the primary exposure and insurance category as the primary enabling-resource predictor, along with demographic and socioeconomic covariates (age group, sex, education, marital status, employment, metro/non-metro residence, low income, and receipt of government assistance). For Hispanic identity–outcome models, we adjusted for age and sex and conditioned on enabling resource variables (insurance, education, income, employment, government assistance, metro), which are plausibly on the pathway from Hispanic identity to outcomes (Figures 2, 3). Accordingly, these models estimate associations conditional on enabling resources rather than a total association. Post-estimation margins are presented to improve interpretability of conditional disparities. Accordingly, these models estimate associations conditional on enabling resources rather than a total association. Post-estimation margins are presented to improve interpretability of conditional disparities. We note in the Limitations that conditioning on mediators may attenuate the total disparity.

**Table 1:** Demographic characteristics for NSDUH sample, 2023 (*n* = 56,705) (weighted).

Variable	Hispanic		Non-Hispanic		Total		*P-value*
	**%**	**SD**	**%**	**SD**	**%**	**SD**	
Demographics							
Age group							< 0.01
12–17 Years old	13%	0.49	8%	0.21	9%	0.20	-
18–25 Years old	16%	0.59	11%	0.24	12%	0.25	-
26–34 years old	17%	0.61	14%	0.25	14%	0.25	-
35–49 years old	26%	0.70	22%	0.35	23%	0.30	-
50–64 years old	18%	0.95	23%	0.49	22%	0.47	-
65 years or older	10%	0.61	23%	0.51	20%	0.42	-
Female	50%	0.97	51%	0.41	51%	0.39	0.16
College	32%	0.80	41%	0.53	40%	0.48	< 0.01
Married	40%	0.91	45%	0.53	44%	0.49	< 0.01
Employment	55%	0.97	55%	0.45	55%	0.39	0.90
Small or rural metro	35%	1.19	47%	0.84	45%	0.80	< 0.01
Income (< $30,000)	27%	0.92	21%	0.49	22%	0.46	< 0.01
Government assistance programs	27%	1.18	20%	0.43	21%	0.47	< 0.01
Health							
Health insurance							
No Insurance	16%	0.58	7%	0.22	8%	0.21	< 0.01
Medicare	12%	0.68	25%	0.52	23%	0.42	< 0.01
Medicaid/CHIP	32%	0.78	19%	0.44	22%	0.44	< 0.01
Military	4%	0.46	6%	0.25	5%	0.23	< 0.01
Private health insurance	46%	0.98	63%	0.51	60%	0.50	< 0.01
AMI or SUD	30%	0.87	34%	0.48	33%	0.40	< 0.01
AMI or SUD treatment	20%	0.73	27%	0.37	25%	0.29	< 0.01
Forgone care - substance use							
No insurance	47%	5.60	28%	3.92	30%	3.70	< 0.01
Family/Friends/Religion acceptance	7%	1.13	16%	3.03	15%	2.70	< 0.01
Insufficient insurance	40%	7.83	32%	4.37	33%	4.16	< 0.01
Didn't think it'd work	8%	3.14	27%	3.45	25%	3.10	< 0.01
Transportation, childcare or appointment times	13%	1.67	25%	3.48	23%	3.21	< 0.01
High cost	40%	8.59	43%	4.18	43%	3.94	< 0.01
Worried what people would think	24%	6.56	42%	3.75	40%	3.45	< 0.01
Sought but didn't receive	1%	0.14	1%	0.08	1%	0.07	< 0.10
Forgone care – Mental health							
No insurance	31%	3.53	27%	1.55	28%	1.36	0.23
Family/Friends/Religion acceptance	20%	2.27	14%	1.06	15%	1.04	< 0.01
Insufficient insurance	33%	3.81	31%	1.71	31%	1.59	0.65
Didn't think it'd work	32%	3.74	28%	1.14	29%	0.97	0.39
Transportation, Childcare or Appointment times	19%	2.66	19%	1.25	19%	1.05	0.83
High cost	52%	3.51	50%	1.74	50%	1.37	0.58
Worried what people would think	31%	2.75	27%	1.48	28%	1.28	0.26
Sought but didn't receive	6%	0.40	6%	0.19	6%	0.16	0.67
Alcohol use disorder	9	0.51	10%	0.25	10%	0.23	< 0.05
Prescription medication treatment	1	0.13	1%	0.09	1%	0.08	0.15
Illicit drug use last year	27	0.99	33%	0.43	32%	0.41	< 0.01
%	18%	-	82%	-	100%	-	-
*N* (unweighted)	11,932		44,773		56,705		

p-values correspond to regression or Chi-squared tests depending on the variable.

**Table 2:** Logistic regression models for AMI/SUD and SUD behaviors for NSDUH sample, 2023.

Past year	(1) Met criteria for AMI/SUD		(2) Received AMI/SUD treatment		(3) Alcohol use disorder		(4) Use of MAT for opioids		(5) Illicit drug use	
***n***	43,637		55,091		55,091		55,091		55,091	
	OR	SE	OR	SE	OR	SE	OR	SE	OR	SE
Hispanic	0.68^***^	(0.03)	0.67^***^	(0.04)	0.78^***^	(0.05)	0.59^*^	(0.16)	0.68^***^	(0.04)
Age group										
12–17 years old	baseline									
18–25 years old	-^++^		1.19^***^	(0.07)	6.27^***^	(0.78)	0.629	(0.35)	3.66^***^	(0.31)
26–34 years old	1.25^***^	(0.07)	1.33^***^	(0.09)	7.19^***^	(0.77)	3.19^**^	(1.66)	4.09^***^	(0.29)
35–49 years old	0.86^***^	(0.03)	1.21^***^	(0.08)	5.75^***^	(0.60)	4.44^***^	(2.24)	3.11^***^	(0.24)
50–64 years old	0.50^***^	(0.03)	0.91	(0.08)	4.03^***^	(0.49)	2.557	(1.50)	2.31^***^	(0.20)
65 or Older	0.22^***^	(0.03)	0.41^***^	(0.05)	1.88^***^	(0.33)	0.866	(0.67)	1.86^***^	(0.23)
Female	0.99	(0.03)	1.78^***^	(0.06)	0.66^***^	(0.03)	0.784	(0.14)	0.89^***^	(0.03)
College	1.03	(0.04)	1.43^***^	(0.06)	1.11^*^	(0.06)	0.28^***^	(0.09)	1.05	(0.05)
Married	0.57^***^	(0.02)	0.72^***^	(0.03)	0.66^***^	(0.04)	0.666	(0.17)	0.64^***^	(0.02)
Employed	0.90^**^	(0.04)	0.93^**^	(0.03)	1.20^***^	(0.06)	1.077	(0.26)	1.19^***^	(0.05)
Small metro or rural	1.07^*^	(0.04)	1.18^***^	(0.05)	0.991	(0.05)	1.44^**^	(0.24)	0.97	(0.04)
Insurance type^+^										
No insurance	0.95	(0.09)	0.58^***^	(0.06)	0.931	(0.15)	0.39^*^	(0.19)	0.90	(0.08)
Medicare	1.24^*^	(0.15)	1.43^***^	(0.18)	1.082	(0.15)	0.679	(0.21)	0.85^*^	(0.07)
Medicaid/CHIP	1.25^***^	(0.10)	1.14^**^	(0.07)	0.933	(0.11)	1.372	(0.47)	1.09	(0.07)
Military Affiliated	1.16	(0.12)	1.35^***^	(0.11)	1.063	(0.18)	0.25^***^	(0.10)	0.83^*^	(0.08)
Private/Employment based	1.01	(0.07)	0.95	(0.06)	0.936	(0.10)	0.51^**^	(0.17)	0.91^*^	(0.05)
Low income (< $30,000)	1.05	(0.05)	0.91^*^	(0.05)	0.972	(0.07)	1.314	(0.29)	0.90^***^	(0.03)
Received government assistance	1.26^***^	(0.06)	1.20^***^	(0.06)	0.978	(0.07)	2.15^***^	(0.56)	1.21^***^	(0.06)
*F*	61.71		58.80		40.33		26.23		51.26	

Exponentiated coefficients; Standard errors in parentheses;

^+^Insurance variables are binary indicators (e.g., Medicare = 1 has Medicare, 0 = does not have Medicare);

^++^for Met Criteria for AMI/SUD the 18–25 group is the baseline given no responses from minors | All models include age and sex.

For Hispanic identity–outcome models, estimates are conditional on enabling resources (insurance, education, income, employment, government assistance, metro/non-metro residence), variables conceptualized as on the pathway from Hispanic identity to outcomes. For insurance–outcome models, Hispanic identity is included as a covariate to account for confounding of insurance–outcome associations.

^*^p < 0.10,

^**^p < 0.05,

^***^p < 0.01.

**Figure 4:**
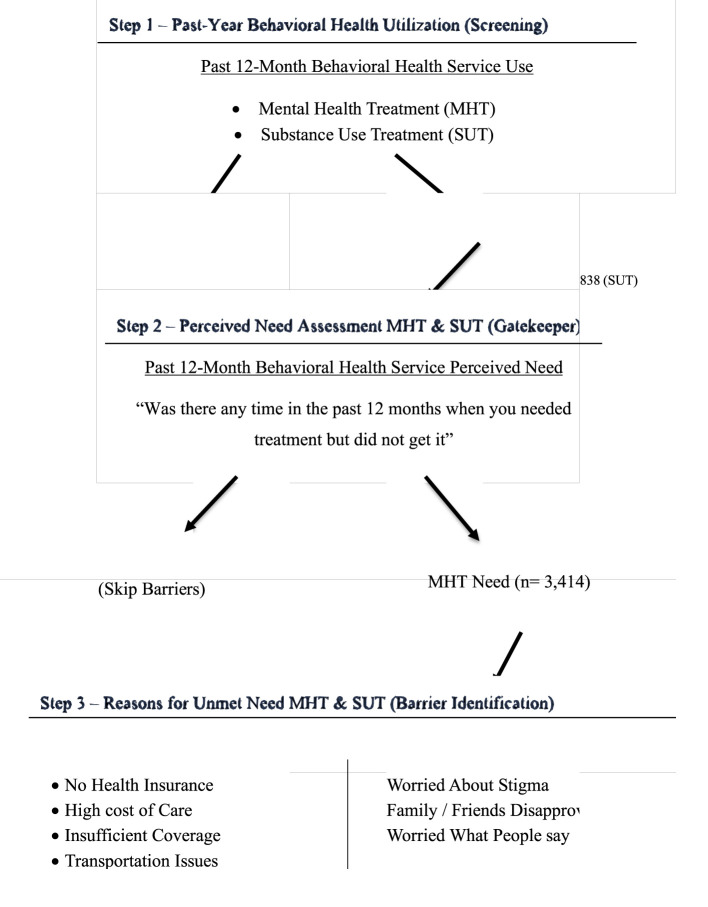
Skip-pattern structure for behavioral health utilization and unmet need. Sample sizes are approximate as item non-response varies.

Insurance was modeled as a set of binary variables (uninsured, Medicare, Medicaid/CHIP, military, private/employer-based) to better reflect real-world overlap and heterogeneity in insurance coverage. For Table 2, we estimated separate logistic regression models for each binary outcome: (1) meeting criteria for AMI or SUD, (2) receipt of any AMI/SUD treatment, (3) alcohol use disorder, (4) use of medication-assisted treatment (MAT) for opioids, and (5) illicit drug use in the past year. Models were estimated on the eligible respondent universe for each outcome (e.g., AMI/SUD defined for adults, and other outcomes defined per NSDUH item eligibility), which explains minor differences in analytic N across columns. For Table 3, the dependent variables were measures of whether the respondent sought substance use or mental health treatment but couldn't get it. For Tables 4, 5, the dependent variables were seven binary indicators representing reasons for forgone care, grouped into attitudinal/stigma-related (“Family, Friends, or Religious Group Wouldn't Like It”; “Didn't Think Treatment Would Help”; “Worried What People Would Think or Say”) and structural/socioeconomic (“No Health Insurance”; “Insufficient Insurance”; “Problem with Childcare, Transportation, or Appointment Times”; “Thought it Cost Too Much”). Separate survey-weighted logistic regressions were estimated for each barrier outcome. These models were restricted to respondents who reported forgone care for the relevant domain, yielding a smaller analytic subsample for each table: substance-use related forgone care barriers (Table 4) and mental-health related forgone care barriers (Table 5). Because the “sought but could not get treatment” item is rare, its analytic N reflects the full eligible domain-specific sample rather than the subset reporting the specific barrier. An illustration of the skip patterns and sample sizes is provided in Figure 4.

**Table 3:** Logistic regression models for seeking care or NSDUH sample, 2023.

Variable	Sought SUD treatment but couldn't get		Sought MH treatment but couldn't get	
***n***	52,098		39,662	
	**OR**	**SE**	**OR**	**SE**
Hispanic	0.52^**^	(0.13)	0.91	(0.08)
Age group				
12–17 years old				
18–25 years old	2.68^**^	(1.02)	1.86^***^	(1.14)
26–34 years old	6.12^***^	(2.26)	1.27^*^	(1.10)
35–49 years old	3.07^***^	(1.25)	0.74^**^	(0.87)
50–64 years old	3.66^***^	(1.75)	0.40^***^	(0.83)
65 or Older	1.82	(0.98)	0.13^***^	(.)
Female	0.92	(0.16)	1.74^***^	(0.28)
College	0.76	(0.15)	1.81^***^	(0.89)
Married	0.579^***^	(0.11)	0.61^***^	(0.22)
Employed	1.00	(0.21)	1.17^*^	(0.30)
Small metro or rural	0.97	(0.17)	1.03	(0.50)
Insurance type				
No insurance	1.39	(0.52)	0.93	(0.47)
Medicare	0.31^***^	(0.10)	1.03	(1.43)
Medicaid/CHIP	0.95	(0.31)	0.74^**^	(0.31)
Military affiliated	1.58	(0.53)	1.24	(0.71)
Private/Employment based	1.21	(0.36)	1.11	(0.47)
Low income (< $30,000)	1.82^**^	(0.42)	0.82^*^	(0.45)
Received government assistance	0.91	(0.20)	1.06	(1.11)
*F*	7.24		58.23	

Exponentiated coefficients; Standard errors in parentheses, | All models include age and sex. For Hispanic identity–outcome models, estimates are conditional on enabling resources (insurance, education, income, employment, government assistance, metro/non-metro residence), variables conceptualized as on the pathway from Hispanic identity to outcomes. For insurance–outcome models, Hispanic identity is included as a covariate to account for confounding of insurance–outcome associations.

^*^p < 0.10,

^**^p < 0.05,

^***^p < 0.01.

**Table 4:** Logistic regression models for forgone care in substance use for NSDUH sample, 2023.

Variable	Attitudinal dependent variables						Structural and socioeconomic dependent variables							
	(1)		(2)		(3)		(4)		(5)		(6)		(7)	
	Family, Friends, or Religious Group Wouldn't Like It		Didn't Think Treatment Would Help		Worried What People Would Think or Say		No Health Insurance		Insufficient Insurance		Problem with Childcare, Transportation, or Appointment Times		Thought it Cost Too Much	
*n*	493		475		501		456		452		495		498	
	**OR**	**SE**	**OR**	**SE**	**OR**	**SE**	**OR**	**SE**	**OR**	**SE**	**OR**	**SE**	**OR**	**SE**
Hispanic	0.36^*^	(0.19)	0.19^***^	(0.08)	0.46^*^	(0.19)	1.45	(0.68)	1.62	(0.77)	0.17^***^	(0.09)	0.82	(0.37)
Age group														
12–17 years old	baseline													
18–25 years old	0.25^*^	(0.19)	1.75	(1.14)	3.62^*^	(2.57)	7.07	(9.66)	1.12	(0.95)	4.52^*^	(3.52)	0.59	(0.41)
26–34 years old	0.28^*^	(0.18)	1.56	(1.10)	2.74	(1.77)	12.84^*^	(17.43)	3.45	(3.15)		(4.96)	0.65	(0.42)
35–49 years old	0.21^**^	(0.15)	1.26	(0.87)	3.49^*^	(2.30)	5.11	(7.21)	1.68	(1.50)	3.76^*^	(2.75)	0.46	(0.33)
50–64 years old	0.33	(0.27)	0.94	(0.83)	1.70	(1.25)	4.68	(6.57)	2.11	(1.92)	1.865	(1.60)	0.39	(0.30)
65 or Older	0.96	(1.06)	1	(.)	2.14	(2.43)	0.40	(0.80)	8.03	(10.08)	10.99^*^	(14.17)	2.66	(3.54)
Female	1.09	(0.53)	0.97	(0.28)	2.30^***^	(0.69)	1.62	(0.50)	3.27^***^	(1.13)	1.28	(0.47)	2.20^**^	(0.81)
College	0.94	(0.43)	2.14^*^	(0.89)	2.39^**^	(0.87)	0.65	(0.22)	0.28^***^	(0.13)	0.73	(0.34)	0.34^***^	(0.13)
Married	0.50	(0.37)	0.42	(0.22)	0.57	(0.22)	1.38	(0.68)	1.15	(0.55)	1.31	(0.66)	1.03	(0.47)
Employed	1.82	(0.83)	0.86	(0.30)	0.80	(0.30)	1.97	(0.82)	2.11	(1.09)	0.38^**^	(0.16)	2.08^**^	(0.71)
Small metro or rural	1.86^*^	(0.67)	1.47	(0.50)	1.09	(0.31)	0.75	(0.35)	0.97	(0.33)	0.78	(0.27)	1.38	(0.42)
Insurance type														
No insurance	1.24	(1.30)	0.61	(0.47)	0.82	(0.59)	1.74	(1.52)	0.58	(0.44)	0.36	(0.33)	0.77	(0.56)
Medicare	0.55	(0.51)	1.09	(1.43)	0.30	(0.24)	0.09^**^	(0.09)	0.36	(0.31)	0.06^**^	(0.06)	0.32	(0.29)
Medicaid/CHIP	0.92	(0.87)	0.47	(0.31)	2.53	(1.56)	0.37	(0.22)	0.52	(0.30)	0.61	(0.36)	1.18	(0.88)
Military Affiliated	0.76	(0.68)	0.77	(0.71)	2.07	(1.61)	0.05^***^	(0.05)	0.38	(0.40)	0.57	(0.46)	0.01^***^	(0.01)
Private/Employment based	0.79	(0.81)	0.67	(0.47)	0.85	(0.54)	0.18^***^	(0.10)	0.45	(0.25)	0.18^***^	(0.08)	0.84	(0.53)
Low Income (< $30,000)	1.77	(0.79)	0.94	(0.45)	0.63	(0.31)	3.63^***^	(1.74)	3.55^**^	(1.73)	1.46	(0.56)	1.80	(0.73)
Received government assistance	0.69	(0.26)	1.98	(1.11)	0.42^**^	(0.17)	0.40^*^	(0.19)	0.18^***^	(0.08)	2.015^*^	(0.76)	0.37^*^	(0.18)
*F*	1.51		3.54		2.51		5.20		5.95		2.77		2.40	

Exponentiated coefficients; Standard errors in parentheses | All models include age and sex. For Hispanic identity–outcome models, estimates are conditional on enabling resources (insurance, education, income, employment, government assistance, metro/non-metro residence), variables conceptualized as on the pathway from Hispanic identity to outcomes. For insurance–outcome models, Hispanic identity is included as a covariate to account for confounding of insurance–outcome associations.

^*^p < 0.10,

^**^p < 0.05,

^***^p < 0.01.

**Table 5:** Logistic regression models for forgone care in mental health treatment for NSDUH sample, 2023.

Variable	Attitudinal dependent variables						Structural and socioeconomic dependent variables							
	(1)		(2)		(3)		(4)		(5)		(6)		(7)	
	Family, Friends, or Religious Group Wouldn't Like It		Didn't Think Treatment Would Help		Worried What People Would Think or Say		No Health Insurance		Insufficient Insurance		Problem with Childcare, Transportation, or Appointment Times		Thought it Cost Too Much	
*n*	3,336		3,286		3,358		3,131		3,037		3,323		3,348	
	**OR**	**SE**	**OR**	**SE**	**OR**	**SE**	**OR**	**SE**	**OR**	**SE**	**OR**	**SE**	**OR**	**SE**
Hispanic	1.20	(0.20)	0.99	(0.24)	1.02	(0.16)	1.00	(0.20)	1.17	(0.23)	0.94	(0.19)	0.99	(0.17)
Age group														
12–17 years old	baseline											
18–25 years old	0.64^*^	(0.16)	0.78	(0.21)	0.78	(0.17)	6.33^***^	(1.88)	7.33^***^	(2.35)	2.14^***^	(0.52)	4.08^***^	(1.13)
26–34 years old	0.21^***^	(0.06)	0.41^***^	(0.10)	0.38^***^	(0.09)	10.37^***^	(3.47)	10.25^***^	(3.39)	2.05^***^	(0.51)	6.00^***^	(1.57)
35–49 years old	0.25^***^	(0.08)	0.40^***^	(0.11)	0.44^***^	(0.09)	6.90^***^	(2.14)	5.25^***^	(1.74)	1.68^*^	(0.51)	2.69^***^	(0.78)
50–64 years old	0.15^***^	(0.06)	0.30^***^	(0.13)	0.25^***^	(0.10)	5.18^***^	(2.66)	6.71^***^	(3.52)	1.34	(0.62)	2.58^**^	(1.17)
65 or Older	0.07	(0.11)	0.15^**^	(0.13)	0.53	(0.45)	15.55^***^	(10.16)	3.50	(2.97)	0.32	(0.33)	2.63	(1.84)
Female	1.07	(0.19)	0.68^***^	(0.09)	0.73^**^	(0.10)	1.07	(0.15)	1.16	(0.18)	1.76^***^	(0.27)	1.24	(0.17)
College	1.18	(0.24)	1.13	(0.23)	1.29	(0.21)	1.12	(0.23)	1.61^**^	(0.30)	0.96	(0.16)	1.07	(0.19)
Married	0.97	(0.20)	1.00	(0.22)	0.80	(0.14)	0.77	(0.14)	1.07	(0.20)	1.77^***^	(0.34)	0.65^**^	(0.12)
Employed	0.87	(0.18)	1.19	(0.18)	0.69^**^	(0.11)	1.14	(0.20)	1.46^**^	(0.23)	0.96	(0.16)	1.10	(0.20)
Small metro or rural	0.97	(0.13)	0.96	(0.13)	1.14	(0.14)	0.93	(0.15)	0.97	(0.13)	1.47^**^	(0.22)	0.95	(0.12)
Insurance type														
No insurance	1.35	(0.67)	0.68	(0.23)	0.56	(0.19)	10.52^***^	(3.23)	1.51	(0.57)	0.49^*^	(0.18)	3.33^***^	(1.19)
Medicare	1.16	(1.30)	2.23	(1.62)	0.80	(0.59)	0.18^***^	(0.09)	1.12	(0.68)	1.00	(0.68)	0.74	(0.37)
Medicaid/CHIP	1.27	(0.51)	1.06	(0.28)	0.80	(0.19)	2.56^***^	(0.71)	1.24	(0.38)	1.01	(0.36)	1.29	(0.39)
Military affiliated	2.07	(1.13)	0.99	(0.51)	1.15	(0.46)	0.99	(0.46)	0.60	(0.34)	0.456^*^	(0.18)	0.58	(0.23)
Private/Employment based	1.36	(0.53)	0.80	(0.20)	0.85	(0.22)	1.02	(0.31)	1.04	(0.31)	0.40^***^	(0.13)	1.12	(0.29)
Low income (< $30,000)	0.54^**^	(0.13)	0.97	(0.21)	0.65^**^	(0.11)	0.99	(0.20)	0.80	(0.15)	0.95	(0.18)	0.85	(0.17)
Received government assistance	1.36	(0.27)	0.98	(0.19)	1.29	(0.21)	1.13	(0.29)	0.86	(0.17)	1.72^***^	(0.32)	0.77	(0.15)
*F*	6.56		3.64		5.45		15.53		4.66		6.32		7.03	

Exponentiated coefficients; Standard errors in parentheses | All models include age and sex. For Hispanic identity–outcome models, estimates are conditional on enabling resources (insurance, education, income, employment, government assistance, metro/non-metro residence), variables conceptualized as on the pathway from Hispanic identity to outcomes. For insurance–outcome models, Hispanic identity is included as a covariate to account for confounding of insurance–outcome associations.

^*^p < 0.10,

^**^p < 0.05,

^***^p < 0.01.

Model specifications reflect Andersen by conditioning treatment use on need (AMI/SUD) and including enabling resources (insurance, income, assistance, metro), and reflect NIMHD by operationalizing determinants primarily at the individual level of the health-care system and sociocultural domains. Although Andersen's model and the NIMHD framework guided the conceptual domains for measurement, covariates were selected based on epidemiologic rationale as enabling-resource- variables that may mediate the association of Hispanic identify with outcomes. Specifically, age group, sex, educational attainment, marital status, employment, metro/non-metro residence, low income (< $30,000), and receipt of government assistance were included because these factors are plausibly associated with both (i) insurance coverage and ethnicity-related social stratification and (ii) substance use, treatment utilization, and perceived barriers to care. For comparability across outcomes, we applied the same confounder adjustment set in all models; analytic samples varied only due to outcome-specific eligibility and domain restrictions.

The general model form is shown in Equation 1:


$$\begin{array}{l} \log\frac{P\left({outcome\;}_{i}=1\right)}{P\left({outcome\;}_{i}=0\right)}=\;\alpha +\beta_{1}{hispanic\;}_{i}+\beta_{2}insurance\text{\_}type_{i}\\ \;+\gamma X_{i}+\varepsilon \end{array}$$ (1)

Where *Y*_*i*_is the binary outcome or barrier indicator for respondent *i*, and *X*_*i*_is the covariate vector. We report ORs with survey-adjusted SEs; statistical significance was assessed using two-sided tests with α = 0.05 (and we denote *P* < 0.10, *P* < 0.05, *P* < 0.01 in the tables). All weighting and variance procedures followed NSDUH public-use guidance and reporting recommendations for complex sample survey analyses.

We hypothesize that β estimators will indicate:

(1) Lower odds of meeting criteria or AMI or SUD or receiving behavioral health treatment.
(2) Higher levels of forgoing care for mental health and substance use treatment and,
(3) Lower prevalence of alcohol and drug use among Hispanic respondents.

To formally test whether AMI/SUD treatment receipt differed between Hispanic and non-Hispanic respondents, and whether any difference varied by AMI/SUD criteria status, we first fit the primary survey-weighted logistic regression model for treatment receipt that included an interaction between ethnicity and AMI/SUD criteria status. We report the interaction term odds ratio and its *P-value* to quantify effect modification on the multiplicative (OR) scale. We also report stratum-specific odds ratios for Hispanic identification within levels of AMI/SUD criteria status, derived using post-estimation linear combinations. After model estimation, we used postestimation predictive margins to translate the interaction effects into model-adjusted probabilities of receiving AMI/SUD treatment for each joint category of ethnicity (Hispanic vs. non-Hispanic) and AMI/SUD criteria status (meets vs. does not meet), along with 95% confidence intervals computed via the delta method under the survey design; these estimates were graphed to produce the margins plot.

## Results

### Descriptive characteristics

Weighted analyses revealed substantial sociodemographic differences between Hispanic and non-Hispanic respondents (Table 1). A greater proportion of Hispanic individuals were younger, with 71.5% under age 50 compared to 55% of non-Hispanics. College completion (31.9% vs. 41.4%, *P* < 0.001) and marriage (39.8% vs. 44.8%, *P* < 0.001) were less common among Hispanics, but they were more likely to reside in small or rural metro areas (47% vs. 34.6%, *P* < 0.001). Furthermore, Hispanics were more likely to be uninsured (16.4% vs. 6.6%, *P* < 0.001), enrolled in Medicaid/CHIP (31.8% vs. 19.4%, *P* < 0.001), and less likely to have private insurance (46.0% vs. 63.3%, *P* < 0.001). Additionally, more Hispanics reported household incomes under $30,000 (26.9% vs. 21.1%) and participation in government assistance programs (27.1% vs. 19.6%; *P* < 0.001 for both).

Hispanic respondents had lower prevalence of any AMI or SUD (30.1% vs. 33.5%, *P* = 0.003), illicit drug use (27.1% vs. 32.5%, *P* < 0.001), and alcohol use disorder (9% vs. 10%, *P* = 0.036) and self-reported treatment utilization remained significantly lower (20.1% vs. 26.5%, *P* < 0.001). Structural barriers were more commonly reported by Hispanic individuals who had unmet or forgone SUD treatment needs, including lack of insurance (46.9% vs. 28%, *P* < 0.001), and insufficient insurance (39.6% vs. 32%, *P* < 0.001). In contrast, stigma-related barriers were less frequently endorsed, including fear of family disapproval (7.4% vs. 16.3%), concern about others' opinions (23.9% vs. 42.3%), treatment skepticism (7.6% vs. 27.0%), and logistical barriers (12.8% vs. 24.0%; *P* < 0.001 for all). Coefficients for adjustment covariates are reported in the tables for completeness but are not interpreted in the text, consistent with recommendations to avoid over-interpreting conditional associations for control variables (56).

### Multivariable predictors of behavioral health outcomes

Multivariable logistic regression models were used to assess predictors of behavioral health outcomes while adjusting for demographic, socioeconomic, and structural variables, as presented in Tables 2–5.

### Substance use behaviors and outcomes

Compared with non-Hispanic respondents, Hispanic individuals had significantly lower odds of reporting illicit drug use [odds ratio (OR), 0.68; *P* < 0.001], AMI/SUD diagnosis (OR, 0.68; *P* < 0.001), and alcohol use disorder (OR, 0.78; *P* < 0.001) (Table 2). They also had lower odds of receiving behavioral health treatment (OR, 0.67; *P* < 0.001) and using medication-assisted treatment (MAT) for opioid use (OR, 0.59; *P* < 0.10). Insurance status was a strong predictor of treatment access. Uninsured individuals had lower odds of treatment utilization (OR, 0.58; *P* < 0.001) and MAT use (OR, 0.39; *P* < 0.10). In contrast, Medicare beneficiaries had the highest odds of treatment use (OR, 1.43; *P* < 0.001). Military insurance was associated with higher treatment use (OR, 1.35; *P* < 0.001) but lower odds of MAT use (OR, 0.25; *P* < 0.001). Medicaid/CHIP recipients had greater odds of meeting AMI/SUD criteria (OR, 1.26; *P* < 0.001) and utilizing treatment (OR, 1.14; *P* < 0.05). To avoid over-adjustment, we report the total association (age-/sex-adjusted) and the association conditional on enabling resources (i.e., conditioned on insurance, education, income, employment, government assistance, and metro residence) for Hispanic identity; insurance–outcome models adjust for Hispanic identity. Margins (adjusted probabilities) are reported for interpretability.

### Perceived barriers to behavioral health care

Separate logistic models were used to examine predictors of seven perceived barriers to care, categorized into structural/socioeconomic (“No Health Insurance”; “Insufficient Insurance”; “Problem with Childcare, Transportation, or Appointment Times”; “Thought it Cost Too Much”) and attitudinal or stigma-related barriers (“Family, Friends, or Religious Group Wouldn't Like It”; “Didn't Think Treatment Would Help”; “Worried What People Would Think or Say”).

#### Substance use treatment barriers

Hispanic respondents generally reported fewer attitudinal barriers relative to non-Hispanic respondents among those in the eligible universe for substance use–related forgone care. Specifically, Hispanic identity was associated with lower adjusted odds of endorsing concerns that family/friends/religious groups would disapprove (OR = 0.36, *P* < 0.10), believing treatment would not help (OR = 0.19, *P* < 0.001), and worrying about what others would think (OR = 0.46, *P* < 0.10). In contrast, patterns for structural barriers among Hispanics were concentrated in insurance-related and access-related domains. Hispanic identity was associated with lower odds of reporting logistical barriers (childcare/transportation/appointment times) (OR = 0.17, *P* < 0.001) and lower odds of reporting that the respondent sought treatment but could not obtain it (OR = 0.52, *P* < 0.05). Associations between Hispanic identity and other structural barriers (e.g., no insurance, insufficient insurance, or cost) were smaller and/or not statistically distinguishable in adjusted models (Table 4).

Insurance coverage was strongly related to several structural barriers. As expected, individuals with private or military coverage rarely reported “no insurance” as a barrier. Medicare coverage was also associated with lower odds of reporting key structural barriers in this domain, including “no health insurance” and logistical barriers (Table 4).

#### Mental health treatment barriers

Ethnicity was not significantly associated with mental health treatment barriers (Table 5). Uninsured individuals were nearly 10 times more likely to report lack of coverage (OR, 10.52; *P* < 0.001) and had significantly higher odds of citing cost as a barrier (OR, 3.33; *P* < 0.001). Private and military insurance were protective against structural barriers, whereas Medicaid was associated with increased odds of reporting uninsurance (OR, 2.56; *P* < 0.001). In contrast, Medicare was linked to lower odds of structural/socioeconomic and attitudinal barriers.

#### Effect modification of Hispanic identification on past-year AMI/SUD treatment by AMI/SUD criteria status

In survey-weighted logistic regression models for past-year AMI/SUD treatment, there was evidence that the association between Hispanic identification and treatment receipt differed by AMI/SUD criteria status (interaction OR, 1.28; *P* = 0.037) (Table 6). Among adults who did not meet AMI/SUD criteria, Hispanic adults had 37% lower odds of receiving AMI/SUD treatment compared with non-Hispanic adults (aOR, 0.63; *P* < 0.001). Among adults meeting AMI/SUD criteria, the disparity was smaller but remained statistically significant (aOR, 0.81; *P* = 0.011).

**Table 6:** Effect modification of Hispanic identification on past-year AMI/SUD treatment by AMI/SUD criteria status.

Contrast from interaction model	Adjusted OR	95% CI	*P-value*
Hispanic vs. non-Hispanic among those who do *not* meet AMI/SUD criteria	0.63	0.52–0.77	<0.001
Hispanic vs. non-Hispanic among those who *meet* AMI/SUD criteria	0.81	0.69–0.95	0.011
Interaction term (Hispanic × AMI/SUD)	1.28	1.01–1.61	0.037

Estimates from survey-weighted logistic regression including covariates for age group, sex, education, employment, metro/rural residence, insurance type, low-income status, and receipt of government assistance. Interaction p-value corresponds to the multiplicative interaction term (Hispanic × AMI/SUD criteria). Stratum-specific odds ratios were computed using post-estimation linear combinations from the interaction model.

**Figure 5:**
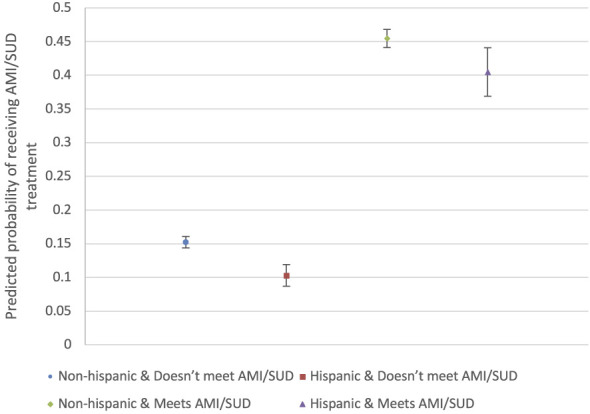
Predicted probability of receiving AMI/SUD treatment by ethnicity and AMI/SUD criteria status. Predicted probabilities are estimated from the survey-weighted logistic regression model including a Hispanic × AMI/SUD interaction and represent adjusted estimates at the observed covariate distribution. Error bars represent 95% confidence intervals. “Meets AMI/SUD” indicates respondents meeting criteria for AMI/SUD; “Doesn't meet AMI/SUD” indicates respondents not meeting criteria.

Marginal standardized predicted probabilities (Figure 5) similarly indicated that Hispanic–non-Hispanic differences in treatment vary by criteria status. Hispanic respondents have a consistently lower adjusted probability of treatment receipt than non-Hispanic respondents both among those meeting criteria and among those not meeting criteria (Figure 5).

## Discussion

This study provides insights into systemic and structural barriers associated with behavioral healthcare access and utilization. Analysis revealed intersections among demographic, structural, and economic factors. These findings are consistent with prior research documenting gender-related disparities in access and utilization (9, 34), suggesting that structural and economic factors may contribute to these patterns.

Uninsured individuals were substantially more likely to cite cost and insurance status as barriers, while Medicare, private, and military coverage were generally associated with fewer perceived barriers. Although Medicaid coverage was associated with higher odds of treatment utilization compared to those without it, many respondents still reported lacking insurance, which may suggest complexities in coverage continuity or awareness, though these factors were not directly measured in this study. These patterns align with prior work on insurance-related disparities (10, 12, 16) and highlight the need for further research on how policy changes influence access.

Targeted analysis provided critical insights into disparities in SUD treatment among Hispanic populations. Although Hispanic respondents reported lower prevalence of illicit drug use, alcohol use disorder, and AMI/SUD, consistent with prior research (8, 29), their treatment utilization was disproportionately low relative to need. For example, despite lower prevalence, Hispanics exhibited significantly reduced odds of receiving behavioral health treatment (OR = 0.67) and medication-assisted treatment (MAT) (OR = 0.59), even after adjusting for clinical need and socioeconomic factors (6, 9, 30, 35).

This suggests that the gap cannot be explained solely by lower prevalence and points to systemic barriers limiting access. Structural and financial barriers, including lack of insurance, high cost, and insufficient coverage, were predominant among Hispanic respondents, whereas stigma-related concerns were less frequently reported. These patterns indicate that treatment disparities are more closely tied to systemic constraints and the lack of enabling resources than to individual reluctance, adding nuance to assumptions that stigma alone accounts for low utilization rates (8, 10, 12, 36, 47, 57). These outcomes reflect the cumulative effect of policy decisions that prioritize punitive frameworks over public health-oriented service delivery. Andersen's Behavioral Model and the NIMHD Research Framework offer complementary lenses for interpreting these patterns; they highlight how “enabling resources” are not randomly distributed but are racially stratified by upstream determinants, including discriminatory insurance designs and the chilling effects of immigration surveillance (58, 59).

These patterns may indicate disparities in the distribution of enabling resources, which are consistent with structural barriers documented in prior research. While our findings cannot establish causation, they underscore the need to consider how policy environments and system-level factors influence access to timely and appropriate behavioral health services. Importantly, our analysis adjusted for clinical need (AMI/SUD) when examining treatment utilization, ensuring that observed disparities are not solely attributable to differences in prevalence. Even among individuals with identified need, Hispanic respondents exhibited significantly lower odds of treatment utilization, suggesting that structural barriers persist beyond differences in need.

### Policy implications

These findings are consistent with concerns that structural inequities in insurance design and delivery may limit access to evidence-based treatment services, including medication-assisted treatment MAT, and other core components of the health service continuum. Policy proposals such as the SUPPORT Act, have expanded access to MAT and telehealth services through Medicaid and Medicare (60). Recent news reports surrounding federal proposals to cut Medicaid by $2.3 trillion and reduce funding to SAMHSA and CDC addiction programs could have implications for MAT access, telehealth-based interventions, and disparities in this study (61–64). While some measures offer promise, such as the expansion of telehealth buprenorphine induction (60), they cannot compensate for the service losses expected under current policy proposals. Further cuts may exacerbate disparities for underserved and racial/ethnic groups, particularly Hispanics, who in this study face diminished access to care due to structural rather than attitudinal barriers. Given that Hispanic respondents in this study were more likely to be uninsured or covered by Medicaid/CHIP, and that this coverage types were associated with higher structural barriers to care, increasing provider network capacity within public insurance programs may help reduce the access constraints disproportionately experienced by these populations.

These concrete measures could help sustain progress achieved under Medicaid expansion and strengthen the overall treatment infrastructure available to populations most affected by structural barriers and social policy retrenchment (65). Future policy efforts should prioritize addressing the barriers reported by Hispanic adults and by individuals who are uninsured or insured through public insurance programs, as these groups demonstrated the highest levels of structural access barriers in this study. Understanding these specific “pain points” can inform targeted interventions that reduce structural barriers where they are most consequential.

### Limitations

This study relied on secondary analysis of publicly available NSDUH data, which, while nationally representative, excludes individuals experiencing institutionalization and homelessness, populations highly vulnerable to SUD and mental illness. Additionally, all measures are self-reported and may be subject to recall and social desirability bias, particularly for stigmatized behaviors such as substance use and attitudes toward treatment. These biases could lead to underreporting of substance use and misrepresentation of perceived barriers, which should be considered when interpreting findings. Furthermore, the cross-sectional nature of the data limits causal inferences, and the absence of longitudinal follow-up prevents understanding long-term treatment outcomes. Because enabling resource variables are plausibly downstream of Hispanic identity, conditioning on them yields associations conditional on these resources and may attenuate total disparities; results should not be interpreted as the causal effect of Hispanic identity independent of downstream pathways-resource variables are plausibly downstream of Hispanic identity, conditioning on them yields associations conditional on these resources and may attenuate total disparities; results should not be interpreted as the causal effect of Hispanic identity independent of downstream pathways-resource variables are plausibly downstream of Hispanic identity, conditioning on them yields associations conditional on these resources and may attenuate total disparities; results should not be interpreted as the causal effect of Hispanic identity independent of downstream pathways.

Although our analysis incorporated indicators of treatment need, the inclusion of respondents without identified need could still obscure patterns of unmet need among high-risk groups. Consequently, the observed lower treatment rates among Hispanics could reflect lower disorder prevalence rather than true disparities in access. Lastly, a critical limitation concerns the aggregation of the “Hispanic” variable. Due to data constraints within publicly available files, this study analyzes Hispanics as a monolithic group, obscuring the substantial heterogeneity within the population. As distinct subgroups (e.g., Puerto Rican, Mexican, Cuban) possess unique sociopolitical histories, legal statuses, and health profiles, our findings cannot be generalized to every specific subpopulation and may mask important intragroup disparities. Furthermore, critical structural determinants such as immigration status, acculturation levels, and detailed geographic location were not available in the dataset and likely act as unobserved confounders in our analysis of treatment access.

## Conclusion

Persistent disparities in substance use treatment may be influenced by systemic factors that affect harm reduction, culturally competent care, and access to evidence-based services. Policy efforts that expand Medicaid and Medicare behavioral health coverage, safeguard funding for MAT and counseling, and improve access for low-income communities may help address these disparities. Strengthening community-based education such as bilingual navigation services and culturally tailored health literacy efforts have been found in prior studies to enhance early intervention, reduce mistrust, and support harm reduction uptake.

However, true equity requires socio-structural interventions that extend beyond the clinic. Future frameworks for care should incorporate the Citizenship Model, which emphasizes the “5 Rs” of rights, responsibilities, roles, resources, and relationships to support full social inclusion for marginalized populations (72). Similarly, adopting Swarbrick's Eight Dimensions of Wellness (financial, environmental, spiritual, etc.) allows for a holistic approach that addresses the economic and environmental deficits identified in this study (66). By aligning behavioral health treatment with these broader socio-structural supports (e.g., housing stability, employment, and legal aid), public health systems can bridge the gap between treatment eligibility and true accessibility.

Future research could incorporate longitudinal data and community-engaged methods to explore the long-term impacts of treatment interventions and the sociopolitical mechanisms that shape stigma and access. Incorporating qualitative insights from Hispanic populations may deepen understanding of how policy, rhetoric, and enforcement practices relate to care-seeking behaviors and institutional barriers.

Equitable access to behavioral health services is widely recognized as an important component of national strategies to address the overdose crisis and promote equity in substance use policy. Expanding access to evidence-based care across all communities, regardless of ethnicity or immigration status, has been identified in prior research as a key approach to advancing health equity and addressing substance use disparities.

## Data Availability

Publicly available datasets were analyzed in this study. This data can be found here: https://nsduhweb.rti.org/respweb/homepage.cfm.
